# Non-Destructive Permittivity and Moisture Analysis in Wooden Heritage Conservation Using Split Ring Resonators and Coaxial Probe

**DOI:** 10.3390/s25164947

**Published:** 2025-08-10

**Authors:** Erika Pittella, Giuseppe Cannazza, Andrea Cataldo, Marta Cavagnaro, Livio D’Alvia, Antonio Masciullo, Raissa Schiavoni, Emanuele Piuzzi

**Affiliations:** 1 Department of Information Engineering, Electronics and Telecommunications, Sapienza University of Rome, 00184 Rome, Italy; marta.cavagnaro@uniroma1.it (M.C.); emanuele.piuzzi@uniroma1.it (E.P.); 2 Institute of Heritage Science National Research Council, 73100 Lecce, Italy; giuseppe.cannazza@unisalento.it; 3 Department of Engineering for Innovation, Complesso Ecotekne-Corpo O, University of Salento, 73100 Lecce, Italy; andrea.cataldo@unisalento.it (A.C.); antonio.masciullo@unisalento.it (A.M.); raissa.schiavoni@unisalento.it (R.S.); 4 Department of Mechanical and Aerospace Engineering, Sapienza University of Rome, 00184 Rome, Italy; livio.dalvia@uniroma1.it

**Keywords:** split ring resonator, antipodal Vivaldi antenna, dielectric permittivity, wireless sensing, cultural heritage monitoring, non-invasive measurement

## Abstract

This study presents a wireless, non-invasive sensing system for monitoring the dielectric permittivity of materials, with a particular focus on applications in cultural heritage conservation. The system integrates a passive split-ring resonator tag, electromagnetically coupled to a compact antipodal Vivaldi antenna, operating in the reactive near-field region. Both numerical simulations and experimental measurements demonstrate that shifts in the antenna’s reflection coefficient resonance frequency correlate with variations in the dielectric permittivity of the material under test. A calibration curve was established using reference materials—including low-density polyvinylchloride, polytetrafluoroethylene, polymethyl methacrylate, and polycarbonate—and validated through precise permittivity measurements. The system was subsequently applied to wood samples (fir, poplar, beech, and oak) at different humidity levels, revealing a sigmoidal relationship between moisture content and permittivity. The behavior was also confirmed using a portable and low-cost setup, consisting of a point-like coaxial sensor that could be easily moved and positioned as needed, enabling localized measurements on specific areas of interest of the sample, together with a miniaturized Vector Network Analyzer. These results underscore the potential of this portable, contactless, and scalable sensing platform for real-world monitoring of cultural heritage materials, enabling minimally invasive assessment of their structural and historical integrity. Moreover, by enabling the estimation of moisture content through dielectric permittivity, the system provides an effective method for early detection of water-induced deterioration in wood-based heritage items. This capability is particularly valuable for preventive conservation, as excessive moisture—often indicated by permittivity values above critical thresholds—can trigger biological or structural degradation.

## 1. Introduction

The conservation of cultural heritage includes a broad range of actions aimed at prolonging the lifespan of artistic and historical artifacts [1]. Within the field of cultural property, conservation efforts are not only directed at preserving the physical integrity of objects but also at maintaining their cultural and historical significance. This dual objective ensures that the intrinsic and extrinsic value of heritage items remains intact over time [2]. A growing area of interest in the field of heritage conservation involves the development of non-invasive, remote monitoring technologies that can assist in the ongoing evaluation of artworks and structural elements such as paintings, wooden frameworks, and stone surfaces. The integration of low-cost wireless systems for remote sensing has emerged as a promising approach due to its scalability, ease of deployment, and minimal impact on cultural heritage materials [3].

In this context, Moisture Content (MC) represents a key-parameter in the conservation field [4], where uncontrolled levels can lead to alterations and accelerating decay caused by chemical and biological processes in different materials (e.g., stones and wood) [5,6]. Literature highlights that in the 0–25% moisture content range—namely Fiber Saturation Point (FSP)—water is primarily absorbed into the wood cell walls. Beyond this threshold, the cell walls are fully saturated, and additional moisture accumulates in the cell cavities. A gravimetric moisture content of approximately 140% corresponds to the Water Saturation Point (WSP). Above the FSP, variations in moisture content significantly influence the physical, mechanical, and rheological properties of wood—such as swelling, shrinkage, stiffness, and strength. Additionally, when moisture content exceeds 5%, the risk of insect infestation notably increases [7,8,9].

In the literature, common methods for measuring MC include Near-Infrared Reflectance Spectroscopy (NIRS) [10], thermography [11,12], Nuclear Magnetic Resonance (NMR) [13], ultrasound [14,15,16], and the Ground Penetrating Radar (GPR) [17], which provide gradient, rather than point-specific, data. Electrical methods—based on resistivity, conductivity, and permittivity (*ε_r_*)—offer a practical balance of resolution, cost, and invasiveness [18]. High-frequency techniques (0.3–300 GHz), such as waveguides and open-ended probes, detect MC via dielectric perturbations but may be affected by temperature and material properties [19]. While waveguides are costly, recent low-cost probes show promise for field use [20]. Low-frequency conductivity-based methods, though simple, are less accurate beyond the FSP due to sensitivity to temperature, salts, and heterogeneity [21]. Capacitance methods, using contact probes at low frequencies, detect ε_r_ variations and provide ~3% accuracy up to the FSP, though density and structural discontinuities can affect results [22].

Among the various sensing technologies under exploration, planar microwave sensors based on resonant structures have attracted considerable attention [23]. These sensors are valued for their compact form factor, high sensitivity, robustness, and ability to detect changes in material properties. The sensing principle is typically based on monitoring shifts in the resonance frequency when a resonator is brought into proximity with a material under test (MUT), which alters the electromagnetic field distribution [24,25]. In particular, split ring resonators (SRRs) have demonstrated significant potential in sensing applications [26,27]. Their popularity derives from their simple and compact geometry, ease of fabrication, and capacity to operate effectively across a range of dielectric materials [28,29]. SRRs have been successfully used in various domains, including material characterization, chemical sensing, and environmental monitoring [29,30].

In this study, we build upon a previously developed design of a groundless SRR sensor tag, originally presented in [31], which was optimized for low-cost fabrication and effective electromagnetic (EM) coupling. While the prior work primarily focused on the design and simulation of the SRR structure, the present study shifts the emphasis toward experimental validation. Specifically, we investigate the use of a lightweight, portable antenna system—based on a compact antipodal Vivaldi antenna—electromagnetically coupled to the SRR tag. The main objective is to extract experimental calibration curves that relate the resonance frequency shift in the antenna’s reflection coefficient (S_11_) to the dielectric permittivity of the MUT. This wireless and contactless sensing approach enables permittivity estimation based on changes in the resonant behavior of the system. Although similar remote sensing configurations have been explored for liquid samples [32,33], this work extends the concept to solid materials relevant in the context of cultural heritage monitoring, with particular focus on practical, real-world implementation using a portable setup.

To further demonstrate the versatility of the system, a complementary sensing configuration was also considered and based on a truncated coaxial probe [34], specifically designed for portable and localized permittivity measurements. This point-like sensor can be easily repositioned across different areas of the sample surface, allowing targeted investigations where degradation or structural anomalies are suspected. This sensor was tested using a miniaturized Vector Network Analyzer (VNA), enabling low-cost, in situ measurements. This method proves suitable for future use as a portable reference tool for localized, in situ permittivity measurements, especially in contexts where cultural heritage materials require minimally invasive diagnostics. While the SRR-Vivaldi configuration provides a fully wireless and passive solution, the coaxial sensor enhances flexibility and spatial resolution, making the combined approach highly suitable for non-invasive assessments of cultural heritage materials.

The paper is organized as follows: Section 2 describes the operational concept at the base of EM simulations and the SRR sensor system, the reference materials used for the experimental calibration curve, the combined uncertainty evaluation, and the complementary sensing element. Section 3 shows the experimental calibration curve obtained by measuring the reference materials. Results on wood samples are shown in Section 4, while conclusions are drawn in Section 5.

## 2. Methods and Models

In this section, the operational concept underlying the EM simulations is illustrated, along with reference materials used to obtain the practical calibration curve. Additionally, the evaluation of measurement uncertainty is also included. Eventually, a specific sensing element, based on truncated coaxial configuration, was also resented.

### 2.1. Operational Concepts and Simulations

**Figure 1:**
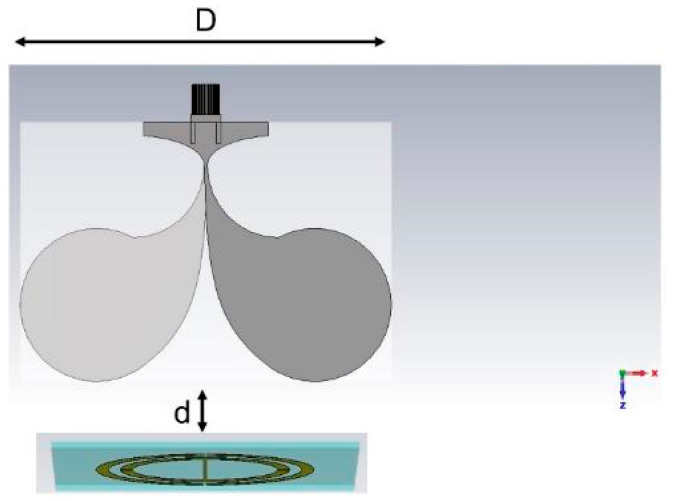
Sensor system schematic with the antipodal Vivaldi antenna coupled with a passive split ring resonator.

The sensor system (Figure 1) includes a passive SRR whose design is presented in [31] and an interrogating antipodal Vivaldi antenna. Since the final idea is to use the system with a portable and low-cost VNA (typical band not exceeding 3 GHz), we concentrated the study of the resonator modes below this frequency. Two principals’ modes around 0.9 GHz and 1.7 GHz were found. The circular groundless planar SRR tag was placed closer than the Fraunhofer distance (d_F_) from the antenna [35], i.e., at R1≪2D2λ (where *D* is the greatest dimension of the antenna, and *λ* is the wavelength at the working frequency). Moreover, to guarantee the operation of the sensor in the reactive near-field region of the antipodal Vivaldi antenna, the distance between the SRR and the antenna is less than R2<0.62×D3λ [36]. Indeed, in the reactive near-field region, the antenna’s field is highly sensitive to electromagnetic absorption, influencing the antenna’s input impedance.

**Figure 2:**
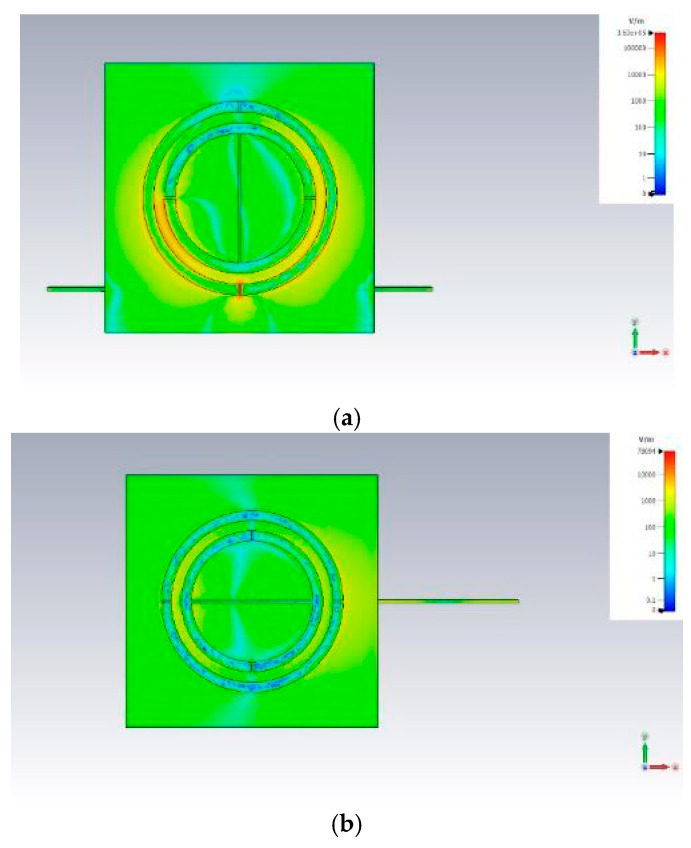
Simulated electric field obtained in the plane of the SSR in the case in which the gap is aligned with (**a**) and perpendicular to (**b**) the antenna electric field polarization.

As illustrated in Figure 2, adjusting the orientation between the SRR gap and the antenna’s electric field polarization allows the ring resonator to function either as a region of strong or minimal electric field intensity. The ring gap is the most responsive area of the tag, making it particularly sensitive to changes in environmental permittivity or the presence of different materials. Figure 2 depicts two distinct alignments of the passive ring tag relative to the antenna’s propagated electric field: one parallel and the other perpendicular. CST Studio Suite^®^ (2023) simulation results revealed that the electric field intensity was significantly higher, especially within the ring gap, when the gap was aligned parallel to the electric field (Figure 2a) compared with the perpendicular configuration (Figure 2b).

Variations in the electric field around the ring structure influence the tag’s electrical characteristics, which in turn alter the antenna’s reflection coefficient due to electromagnetic coupling between the two components. Indeed, the SRR resonance frequency and amplitude were highly responsive to the dielectric properties of the surrounding environment, specifically permittivity and loss tangent, allowing for wireless sensing through the interaction between the antenna and the tag.

**Figure 3:**
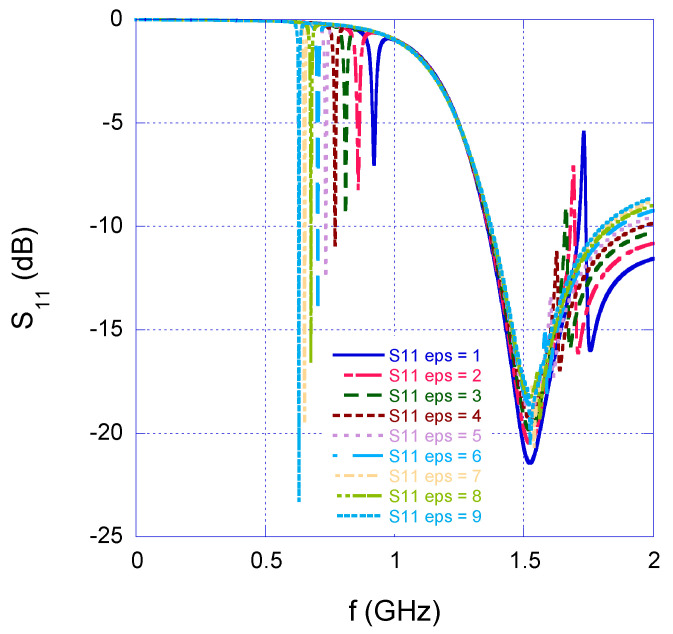
Simulated S_11_ of the antipodal Vivaldi antenna in the presence of the SRR, and the material under test with variable permittivity.

Figure 3 illustrates the simulated reflection coefficient (S_11_) of the antenna when the SRR was positioned within its reactive near-field region. In this scenario, the ring gap was aligned with the electric field polarization, and a material was positioned near the gap. As the material permittivity increased, a downward shift in the resonance frequency was observed. A similar set of CST simulations was conducted using the same passive SRR and material, but this time with the ring gap oriented perpendicular to the electric field: in this configuration no significant frequency shift occurred, indicating that the perpendicular alignment was not sensitive to permittivity variations in the same way. In conclusion, the simulations confirm that by evaluating the resonance frequency of the antenna placed near the SRR acting as a tag, the dielectric properties of the material under test can be monitored.

### 2.2. Reference Materials

The material samples used as a reference are shown in Table 1. In particular, these include the low-density polyvinylchloride (LD-PVC), polytetrafluoroethylene (PTFE), polymethyl methacrylate (PMMA), and polycarbonate (PC) previously characterized in [30] and polyamide 6 (PA6) containing 1%, 3%, and 5% by weight of Graphene Nanoplatelets (GNPs) measured in [37].

Their transverse dimensions allow for insertion into the WR430 waveguide, which has internal dimensions of 109.22 mm × 54.61 mm, enabling proper characterization.

### 2.3. Combined Uncertainty Evaluation

To provide a rigorous metrological assessment of the sensing system, the combined uncertainty in the estimation of the dielectric permittivity (*ε_r_*) was evaluated. The analysis considers both Type A uncertainty (statistical variation from repeated measurements) and Type B uncertainty (systematic effects such as calibration model error and instrument resolution). Given a set of *n* permittivity values, *ε_ri_*, derived from repeated resonance frequency (*f_r_*) measurements on different points of a wood sample at a fixed humidity level, the total combined uncertainty was computed using the following expression:

(1) $$u_{c\left(\varepsilon_{r}\right)}=\sqrt{{\left(\frac{s}{\sqrt{n}}\right)}^{2}+\;u_{B,cal}^{2}+\;{\left(\left|\frac{{d\varepsilon }_{r}}{{df}_{r}}\right|\times \;\sigma_{f,VNA}\right)}^{2}\;}$$

where *s* is the standard deviation of the *ε_r,i_* values (Type A uncertainty), *n* is the number of repeated measurements, *u_B,cal_* is the uncertainty associated with the empirical calibration curve *ε_r_ = f(f_r_)*, *σ_f,VNA_* is the frequency accuracy of the vector network analyzer (VNA), and *dε_r_/df_r_* is the derivative of the calibration curve with respect to frequency *f*.

According to the official datasheet of the PNA E8363C (Agilent Technologies, Santa Clara, CA, USA) [38], the frequency accuracy is generally of the order of

(2) $$\sigma_{\mathrm{f,VNA}}\;\approx \;\pm (0.5\;\mathrm{ppm}\cdot f+1\;\mathrm{Hz})$$

### 2.4. Complementary Portable Sensing System

**Figure 4:**
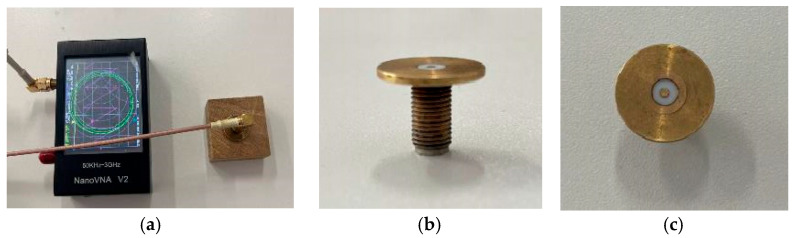
Experimental setup based on the truncated coaxial probe and miniaturized VNA. (**a**) Measurement configuration during acquisition on a wood sample using the NanoVNA device (HCXQS, Nanjing, China). (**b**) Lateral view of SE. (**c**) Front view of the SE.

For the sake of assessing the aforementioned methodology and also for proposing a complementary method suitable for in situ, low-cost monitoring application, a specific sensing element (SE), based on truncated coaxial configuration was also used. This SE was connected to a miniaturized Vector Network Analyzer (m-VNA), operating in the 1–3 GHz frequency range so as to measure the complex reflection coefficient (S_11_) of the material under test (see Figure 4). The advantages of using a m-VNA over a benchtop VNA are mainly related to its size (dimensions 15 cm × 10 cm × 6 cm), cost (about 100 Euros), and versatility. In addition, it has been shown that the metrological performance of this kind of device is comparable to that of advanced bench-top instruments [39].

As for the SE, a truncated open-ended coaxial probe was chosen as the basic configuration and was customized to meet the requirements of non-invasiveness, good surface contact with the material under test, and good spatial resolution. In particular, the outer diameter of the inner conductor is 1.25 mm, the inner diameter of the outer conductor is 4.2 mm, and the dielectric material between the two conductors is Teflon. The probe, having an external length of 11 mm, is equipped with an external metallic flange (i.e., a ground plane), with a diameter of 17.2 mm, in order to enhance its sensitivity in terms of the fringing field effect.

Thanks to its compact size and point-like configuration, the sensing element enables localized dielectric measurements at selected positions of the sample. To extract the complex dielectric permittivity as a function of frequency from the measured S_11_ (f), a custom algorithm was implemented in LabVIEW. This algorithm is specifically designed for customized coaxial probes and does not require full analytical modeling of the probe capacitance. The procedure includes a calibration step based on well-characterized dielectric reference materials chosen for their known frequency-dependent permittivity. This calibration allows compensation for systematic errors and parasitic effects introduced by the measurement setup and the probe geometry. Once calibrated, the system enables reliable, contact-based dielectric characterization of unknown samples across the operational frequency range. In this study, it was applied to one of the wood specimens previously tested with the SRR-based setup, in order to validate the sensing approach.

## 3. Experimental Calibration Curve

In this Section, the calibration curve that links the permittivity with the resonance frequency of the antipodal Vivaldi antenna is evaluated. In particular, the calibration curve has been achieved by measuring the S_11_ of the antenna connected to the PNA Network Analyzer E8363C (10 MHz–40 GHz), Agilent Technologies (Santa Clara, CA, USA). This choice allows the consideration of a more realistic situation with actual samples, reported in Table 1, to extract the calibration curve. In fact, in [31], it was evidenced that the calibration curve was sensitive to the dimensions of the sample and part of the observed error was attributed to the fact that the experimental sample dimensions and features were not perfectly coincident with those of the simulated sample; moreover, the simulations with material samples of representative dimensions were very time consuming.

**Figure 5:**
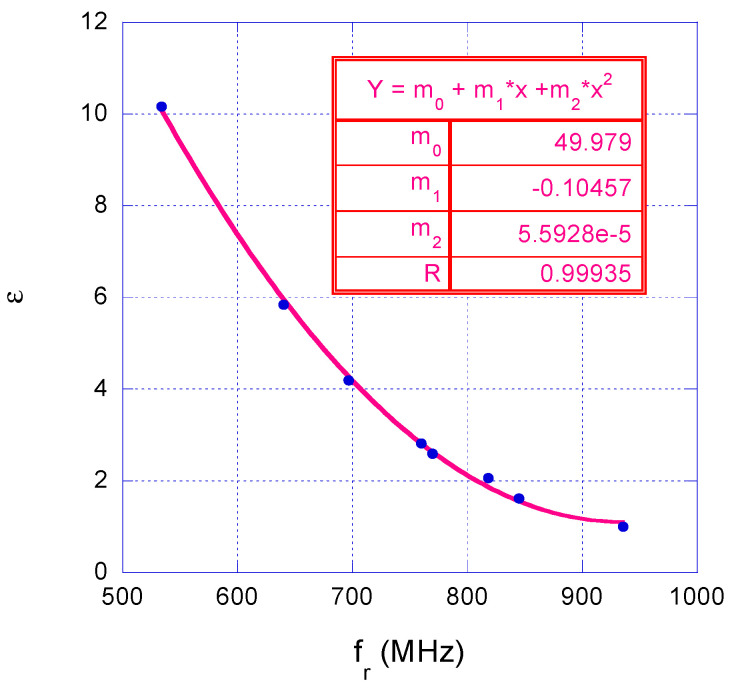
Experimental calibration curve of the wireless sensor system.

The calibration curve obtained by these measurement campaigns is shown in Figure 5 and is given by the following:

(3) $$\varepsilon_{r}=m_{0}+m_{1}\cdot f_{r}+m_{2}\cdot f_{r}^{2}$$

where *f_r_* is the resonance frequency in MHz, m_0_ = 49.979, m_1_ = −0.10457 Hz^−1^, and m_2_ = 5.59·10^−5^ Hz^−2^.

## 4. Measurements on Wood Samples

**Figure 6:**
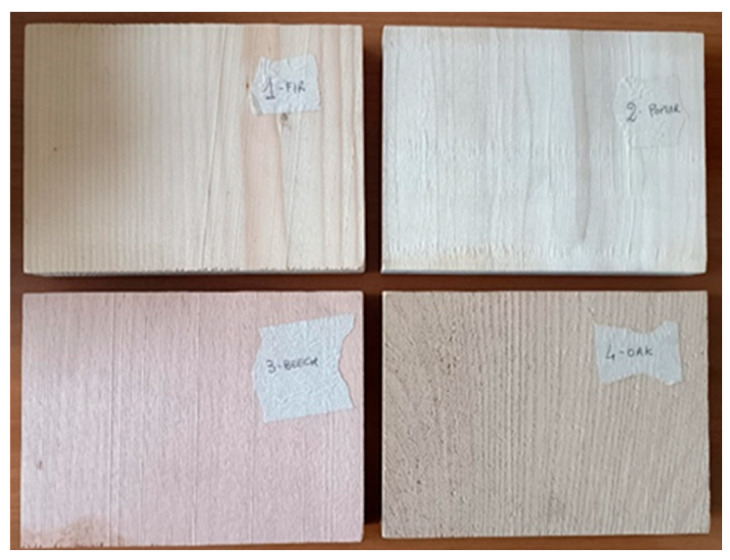
Picture of the wood sample.

Four wood samples were measured with the wireless sensor system: fir, poplar, beech, and oak (Figure 6). All samples have the following transversal dimensions: 14.8 cm ×10 cm × 2 cm.

Initially, the dried sample was weighed using an electronic balance with a 10 mg resolution. The antenna’s response in the presence of the dry sample was then measured using the VNA. Subsequently, the sample was immersed in deionized water for several minutes and later measured at six distinct humidity levels. The gravimetric Moisture Content (θ%) was determined using the following equation:

(4) $$\theta_{g}=\;\frac{W_{i}-W_{dry}}{W_{dry}}\times 100$$

where W_i_ is the mass of the moist specimen, and Wdry is the mass of the dried sample, as suggested in UNI EN 13755 [40]. The steps of the measurement procedure are similar to those detailed in [41]. In particular, for each humidity level of the samples, ten repeated S_11_ measurements were performed positioning the SRR tag in different points of the sample. Therefore, ten values of the resonance frequency have been found for each humidity level, and the mean frequency with the standard deviation has been computed.

**Figure 7:**
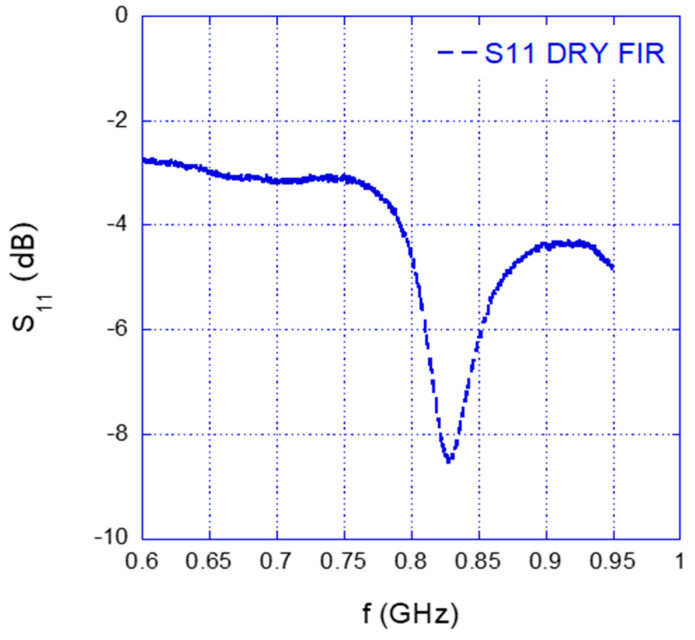
Measured S_11_ of the antenna in front of the SRR placed in contact with the dry fir sample.

Figure 7 shows the reflection coefficient behavior of the antenna in the presence of dry fir wood as an example. Similar trends were observed for the other types of wood. Therefore, it suffices to evaluate the f_r_ mean value and to infer the corresponding value of *ɛ_r_* through the experimental calibration curve (3). The computed mean frequency values with the corresponding standard deviations are reported in Table 2, while Table 3 reports the evaluated values of permittivity and expanded uncertainty.

**Figure 8:**
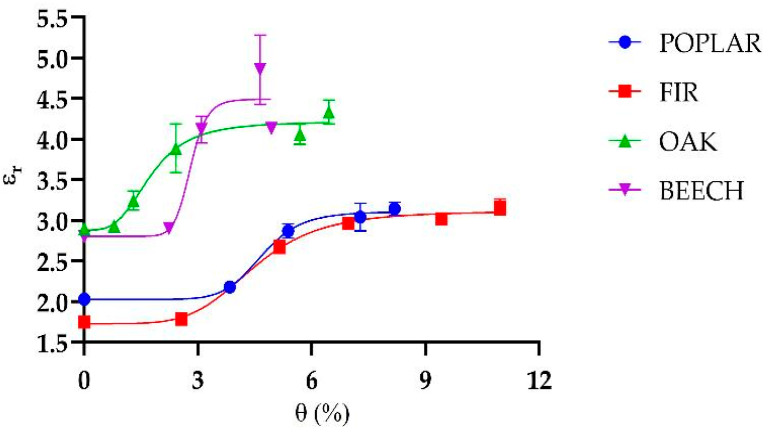
Relationship between wood humidity levels and dielectric permittivity of the wood.

Figure 8 shows that the relationship between permittivity and humidity has a sigmoidal trend. It can be attributed to the way water interacts with the material at different moisture levels. At low humidity, only a small amount of bound water is present, which causes minimal changes in permittivity; in fact, bound water is subject to a number of forces that hinder its response to an imposed EM field resulting in a lower relative permittivity than that of free water [42,43]. As humidity increases, more water is absorbed into the material, including free water, leading to a rapid increase in the dielectric constant due to water’s high permittivity [42,43]. Eventually, the material approaches saturation, and the rate of change in permittivity levels off, resulting in the characteristic S-shaped curve. The sigmoidal trend observed in the relationship between dielectric permittivity and Moisture Content was modeled using the following equation:

(5) $$y\;={\;a}_{1}+\frac{a_{2}-a_{1}}{1+{\left(\frac{a_{3}}{x}\right)}^{a_{4}}}$$

where *y* is the dielectric permittivity, *x* is the water content, and *a*_1_, *a*_2_, *a*_3_, *a*_4_ are the fitting coefficients. The values of these parameters for different wood species are reported in Table 4.

**Figure 9:**
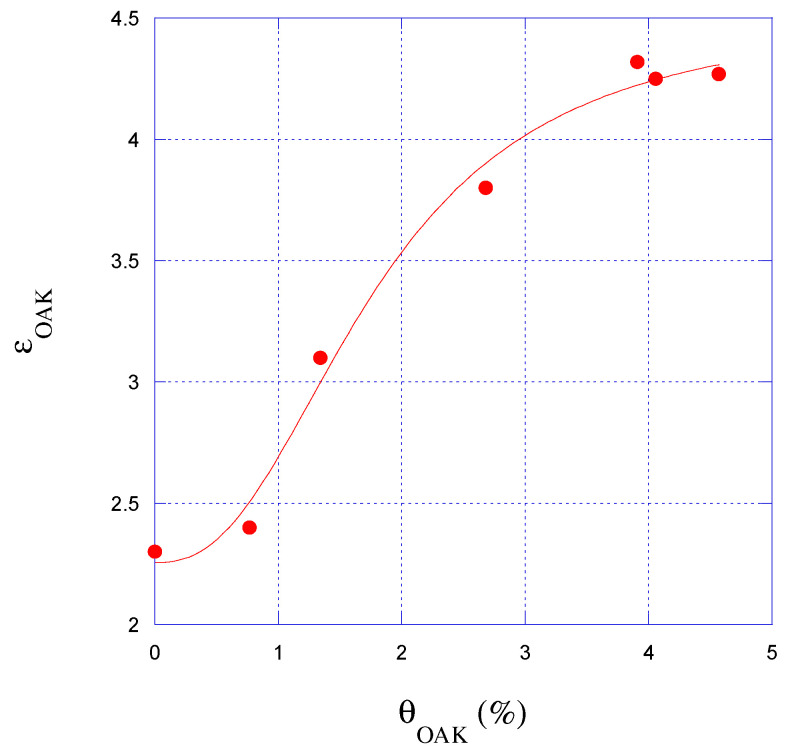
Relationship between oak humidity levels and dielectric permittivity measured with the SE—m-VNA setup.

The oak sample was also analyzed using the SE and the m-VNA setup, described in Section 2.4, for low-cost, remotely controllable in situ monitoring. Although this validation was conducted on a single sample, the results shown in Figure 9 demonstrate a good agreement between the two systems, supporting the feasibility and effectiveness of the proposed approach for practical applications. In particular, the SE-based method proved suitable for performing localized and repeatable reference measurements, especially in real-world scenarios where wireless sensing might not be feasible or where direct contact assessment is required. Despite some differences in the absolute permittivity values—likely due to the non-uniform moisture distribution within the wood—the characteristic sigmoidal trend was clearly observed, further validating the reliability of the measurements.

Moreover, the results are aligned with results reported in the literature, such as in [22], where a sigmoidal relationship between moisture content and permittivity is reported in Figure 4 for a pine sample. As the final point of discussion, the apparent saturation of the permittivity at relatively low MC levels (e.g., 4–12%) is not an intrinsic limitation of the sensor sensitivity, but rather a result of the moisture uptake methodology, which does not ensure uniform or full saturation of the samples.

## 5. Conclusions

This study successfully demonstrated the feasibility of a passive split-ring resonator system coupled with an antipodal Vivaldi antenna for non-invasive, contactless permittivity sensing, with a focus on applications in cultural heritage conservation. The system is based on electromagnetic coupling in the reactive near-field region to detect shifts in the resonance frequency of the antenna’s reflection coefficient, which correlates with changes in the dielectric permittivity of the material under test. Through experimental validation, a robust calibration curve was established, linking resonance frequency shifts to permittivity values for a range of reference materials. Further validation on four wood samples (fir, poplar, beech, and oak) under varying humidity levels revealed a sigmoidal relationship between moisture content and permittivity. This behavior is attributed to the interaction of water molecules with the wood matrix, where bound water at low humidity levels minimally affects permittivity, while free water absorption at higher levels leads to significant dielectric changes. The system’s portability, scalability, and minimal invasiveness make it particularly suitable for real-world monitoring of cultural heritage materials, where preserving structural and historical integrity is paramount. The results underscore the potential of this wireless sensing approach as a practical tool for heritage conservation, enabling continuous and non-destructive monitoring of material degradation. As a complementary contribution, this work also introduced a point-like, contact-based coaxial sensing element (SE) developed by our group and tested in combination with a miniaturized VNA (m-VNA). Additionally, the sigmoidal relationship between moisture content and dielectric permittivity was also confirmed using the SE and m-VNA setup, highlighting the method’s reliability even when implemented with low-cost, portable instrumentation. This secondary method not only provides an effective validation tool for the wireless system but also offers a practical solution for localized in situ reference measurements, especially in contexts where remote sensing is not applicable or precise spatial resolution is required.

Although both sensing methods—the wireless SRR-Vivaldi and contact-based coaxial probe—demonstrated a consistent sigmoidal relationship between permittivity and moisture content, some differences were observed in the absolute permittivity values measured from oak wood, with variations up to 20%. These discrepancies can be attributed to several factors, including the difference in sensing volume (distributed vs. localized), variations in humidity distribution within the sample and the sensitivity of each system to material and environmental conditions. The coaxial probe, being in direct contact with the material and calibrated using well-known standards, offers higher local accuracy and metrological reliability. On the other hand, the SRR-based method enables a non-contact, distributed assessment and is more suitable for rapid and widespread monitoring. Both systems are complementary: the contact method serves as a reliable reference tool, while the wireless sensor enables broader and faster inspection. In the current study, permittivity values obtained from the coaxial probe can be considered more precise for localized measurements, while the wireless setup offers practical advantages for field deployment.

Finally, although our system does not directly provide moisture measurements, the established calibration curve between permittivity and Moisture Content enables non-invasive moisture estimation once the reference sigmoid is defined for each material.

Future research should focus on the use of a portable and low-cost VNA [44], especially considering that the resonator was specifically designed to operate within the frequency range supported by such instruments. Subsequent efforts should aim at extending the methodology to other heritage materials—such as stone, ceramics, and textiles—to validate its broader applicability, enhancing sensitivity and reducing measurement uncertainties through refined SRR and antenna designs, integrating machine learning algorithms for automated permittivity extraction and the early detection of material degradation, evaluating performance under varying temperature and humidity conditions to ensure reliability in diverse conservation settings within a climate chamber to obtain a well-controlled environment, and implementing the system in real heritage sites to assess its long-term durability and effectiveness in practical scenarios.

As a final consideration, despite the system being primarily designed for heritage conservation, the underlying sensing principle may also be adapted to industrial contexts, such as the non-destructive classification of rough sawmill wood, thus opening new frontiers for quality control in wood processing.

## Figures and Tables

**Table 1:** Reference Materials [30,37] used for calibration.

**Material Samples**	$\varepsilon_{r}$	$\delta \varepsilon_{r}$
Air	1.00	-
LD-PVC	1.620	0.003
PTFE	2.060	0.004
PMMA	2.590	0.005
PC	2.820	0.006
PA6-GNP 1%	4.195	0.019
PA6-GNP 3%	5.846	0.004
PA6-GNP 5%	10.172	0.069

**Table 2:** Average frequency values calculated from ten repeated measurements at six different humidity levels. Frequency and standard deviation are expressed in MHz.

FIR	ϑ_1_ = 0.00%		ϑ_2_ = 2.56%		ϑ_3_ = 5.15%		ϑ_4_ = 6.97%		ϑ_5_ = 9.49%		ϑ_6_ = 10.96 %	
	*f_mean_*	*σ_f_*	*f_mean_*	*σ_f_*	*f_mean_*	*σ_f_*	*f_mean_*	*σ_f_*	*f_mean_*	*σ_f_*	*f_mean_*	*σ_f_*
	827.3	2.4	824.0	3.6	767.1	4.4	752.5	3.6	749.5	3.3	742.6	4.5
POPLAR	ϑ_1_ = 0.00%		ϑ_2_ = 3.83%		ϑ_3_ = 5.31%		ϑ_4_ = 7.23%		ϑ_5_ = 8.18%			
	*f_mean_*	*σ_f_*	*f_mean_*	*σ_f_*	*f_mean_*	*σ_f_*	*f_mean_*	*σ_f_*	*f_mean_*	*σ_f_*		
	805.89	1.50	795.86	1.28	756.86	4.37	748.71	8.14	743.74	3.89		
BEECH	ϑ_1_ = 0.00%		ϑ_2_ = 2.24%		ϑ_3_ = 3.09%		ϑ_4_ = 4.64%		ϑ_5_ = 4.94%			
	*f_mean_*	*σ_f_*	*f_mean_*	*σ_f_*	*f_mean_*	*σ_f_*	*f_mean_*	*σ_f_*	*f_mean_*	*σ_f_*		
	760.53	1.79	755.37	1.49	702.605	6.37	643.84	13.32	702.015	2.04		
OAK	ϑ_1_ = 0.00%		ϑ_2_ = 0.79%		ϑ_3_ = 1.29%		ϑ_4_ = 2.42%		ϑ_5_ = 5.69%		ϑ_6_ = 6.45%	
	*f_mean_*	*σ_f_*	*f_mean_*	*σ_f_*	*f_mean_*	*σ_f_*	*f_mean_*	*σ_f_*	*f_mean_*	*σ_f_*	*f_mean_*	*σ_f_*
	755.30	2.08	753.86	1.92	739.13	5.48	711.87	12.03	704.78	4.90	694.42	5.51

**Table 3:** Average permittivity values calculated from ten repeated measurements at six different humidity levels. Permittivity and expanded uncertainty are reported.

FIR	ϑ_1_ = 0%		ϑ_2_ = 2.56%		ϑ_3_ = 5.153%		ϑ_4_ = 6.966%		ϑ_5_ = 9.4854%		ϑ_6_ = 10.96%	
	$\varepsilon_{r}$ *_mean_*	$u_{c\left(\varepsilon_{r}\right)}$	$\varepsilon_{r}$ *_mean_*	$u_{c\left(\varepsilon_{r}\right)}$	$\varepsilon_{r}$ *_mean_*	$u_{c\left(\varepsilon_{r}\right)}$	$\varepsilon_{r}$ *_mean_*	$u_{c\left(\varepsilon_{r}\right)}$	$\varepsilon_{r}$ *_mean_*	$u_{c\left(\varepsilon_{r}\right)}$	$\varepsilon_{r}$ *_mean_*	$u_{c\left(\varepsilon_{r}\right)}$
	1.747	0.057	1.787	0.0593	2.676	0.073	2.960	0.072	3.022	0.0719	3.169	0.079
POPLAR	ϑ_1_ = 0%		ϑ_2_ = 3.838%		ϑ_3_ = 5.3137%		ϑ_4_ = 7.2752%		ϑ_5_ = 8.1861%			
	$\varepsilon_{r}$ *_mean_*	$u_{c\left(\varepsilon_{r}\right)}$	$\varepsilon_{r}$ *_mean_*	$u_{c\left(\varepsilon_{r}\right)}$	$\varepsilon_{r}$ *_mean_*	$u_{c\left(\varepsilon_{r}\right)}$	$\varepsilon_{r}$ *_mean_*	$u_{c\left(\varepsilon_{r}\right)}$	$\varepsilon_{r}$ *_mean_*	$u_{c\left(\varepsilon_{r}\right)}$		
	2.030	0.056	2.181	0.056	2.873	0.069	3.041	0.100	3.143	0.075		
BEECH	ϑ_1_ = 0%		ϑ_2_ = 2.2396%		ϑ_3_ = 3.0885%		ϑ_4_ = 4.6372%		ϑ_5_ = 4.9352%			
	$\varepsilon_{r}$ *_mean_*	$u_{c\left(\varepsilon_{r}\right)}$	$\varepsilon_{r}$ *_mean_*	$u_{c\left(\varepsilon_{r}\right)}$	$\varepsilon_{r}$ *_mean_*	$u_{c\left(\varepsilon_{r}\right)}$	$\varepsilon_{r}$ *_mean_*	$u_{c\left(\varepsilon_{r}\right)}$	$\varepsilon_{r}$ *_mean_*	$u_{c\left(\varepsilon_{r}\right)}$		
	2.780	0.058	2.902	0.057	4.119	0.097	4.855	0.202	4.132	0.069		
OAK	ϑ_1_ = 0%		ϑ_2_ = 0.7915%		ϑ_3_ = 1.2939%		ϑ_4_ = 2.4189%		ϑ_5_ = 5.6914%		ϑ_6_ = 6.4473%	
	$\varepsilon_{r}$ *_mean_*	$u_{c\left(\varepsilon_{r}\right)}$	$\varepsilon_{r}$ *_mean_*	$u_{c\left(\varepsilon_{r}\right)}$	$\varepsilon_{r}$ *_mean_*	$u_{c\left(\varepsilon_{r}\right)}$	$\varepsilon_{r}$ *_mean_*	$u_{c\left(\varepsilon_{r}\right)}$	$\varepsilon_{r}$ *_mean_*	$u_{c\left(\varepsilon_{r}\right)}$	$\varepsilon_{r}$ *_mean_*	$u_{c\left(\varepsilon_{r}\right)}$
	2.903	0.059	2.932	0.059	3.244	0.082	3.889	0.149	4.062	0.086	4.335	0.094

**Table 4:** Parameters of the sigmoid for the 4 measured woods.

**y = *a*_1_ + (*a*_2_ − *a*_1_)/(1 + (*a*_3_/x)^*a*_4_)**				
**Sigmoid Coefficients**	**Poplar**	**Fir**	**Oak**	**Beech**
*a*_1_	2.028	1.730	2.882	2.805
*a*_2_	3.114	4.218	4.494	4.514
*a*_3_	4.677	4.472	1.751	2.800
*a*_4_	8.990	5.050	3.436	12.90
R^2^	0.97	0.98	0.98	0.90

## Data Availability

Data available upon request.
